# Comparative Proteomics of *Coxiella* like Endosymbionts (CLEs) in the Symbiotic Organs of Rhipicephalus sanguineus Ticks

**DOI:** 10.1128/spectrum.01673-21

**Published:** 2022-01-12

**Authors:** Balasubramanian Cibichakravarthy, Juan A. Oses-Prieto, Michael Ben-Yosef, Alma L. Burlingame, Timothy L. Karr, Yuval Gottlieb

**Affiliations:** a Koret School of Veterinary Medicine, The Robert H. Smith Faculty of Agriculture, Food and Environment, The Hebrew University of Jerusalem, Rehovot, Israel; b Department of Pharmaceutical Chemistry, University of California, San Francisco, California, USA; c The Biodesign Institute, Mass Spectrometry Core Facility, Arizona State University, Tempe, Arizona, USA; University of Guelph

**Keywords:** symbiosis, obligate blood feeders, comparative proteomics, gene ontology

## Abstract

Maternally transmitted obligatory endosymbionts are found in the female gonads as well as in somatic tissue and are expected to provide missing metabolite to their hosts. These deficiencies are presumably complemented through specific symbiotic microorganisms such as *Coxiella*-like endosymbionts (CLEs) of *Rhipicephalus* ticks. CLEs are localized in specialized host tissue cells within the Malpighian tubules (Mt) and the ovaries (Ov) from which they are maternally transmitted to developing oocytes. These two organs differ in function and cell types, but the role of CLEs in these tissues is unknown. To probe possible functions of CLEs, comparative proteomics was performed between Mt and Ov of R. sanguineus ticks. Altogether, a total of 580 and 614 CLE proteins were identified in Mt and Ov, respectively. Of these, 276 CLE proteins were more abundant in Mt, of which 12 were significantly differentially abundant. In Ov, 290 CLE proteins were more abundant, of which 16 were significantly differentially abundant. Gene Ontology analysis revealed that most of the proteins enriched in Mt are related to cellular metabolic functions and stress responses, whereas in Ov, the majority were related to cell proliferation suggesting CLEs function differentially and interdependently with host requirements specific to each organ. The results suggest Mt CLEs provide essential nutrients to its host and Ov CLEs promote proliferation and vertical transmission to tick progeny.

**IMPORTANCE** Here we compare the *Coxiella*-like endosymbionts (CLEs) proteomes from Malpighian tubule (Mt) and the ovaries (Ov) of the brown dog tick Rhipicephalus sanguineus. Our results support the hypothesis that CLEs function interdependently with host requirements in each of the organs. The different functional specificity of CLE in the same host suggest that metabolic capabilities evolved according to the constrains imposed by the specific organ function and requirements. Our findings provide specific CLE protein targets that can be useful for future studies of CLE biology with a focus on tick population control.

## INTRODUCTION

Highly successful co-evolution between arthropod hosts and endosymbionts often involve nutrient supplementation to the host by the endosymbiont (1). These endosymbionts are commonly found in specific organs in arthropods with specialized diets deficient in essential nutrients. For obligatory blood feeders such as ticks, the endosymbionts provide their hosts with essential B vitamins lacking in blood and also localized to ovarian tissues to facilitate maternal transmission via the egg (2). A notable example are *Coxiella*-like endosymbionts (CLEs) present in many tick genera including all tested *Rhipicephalus* species (3). CLEs preferentially colonize the ovaries, Malpighian tubules and in some cases also the salivary glands of the ticks, suggesting possible roles in metabolism, fecundity and osmoregulation (4–8). Colonization of the ovaries promotes invasion into the oocyte cytoplasm and enables maternal transmission to the offspring. The dense colonization of Malpighian tubules by CLEs supports a nutritional role for these symbionts, as Malpighian tubules are involved in metabolite excretion and in osmoregulation (9) and can serve as bacteriomes (symbiont hosting organs) in other arthropods (10, 11). Indeed, reduced infection density of CLE through antibiotic treatment resulted in imperfect vertical transmission and major reduction in tick survival and reproduction (12–15).

Aside from experimental evidence establishing a link between symbiotic and aposymbiotic host fitness, the molecular basis for the obligatory association between CLE and ticks are poorly understood. Previous genomic data revealed large reductions in CLE genome size while retaining genetic pathways for synthesis and utilization of compounds essential for the host, such as amino acids, B vitamins, and cofactors (16–19). While this general scenario explains the overall logic of a role for the endosymbiont in essential nutrient provisioning, it has yet to explain endosymbiont tissue distribution or any specific functional roles in Mt and Ov. We therefore undertook a comparative proteomics approach to determine tissue-specific CLE protein distributions and relative abundances in Mt and Ov of *R. sanguineus* ticks.

## RESULTS

### Quantitative protein analysis.

**FIG 1:**
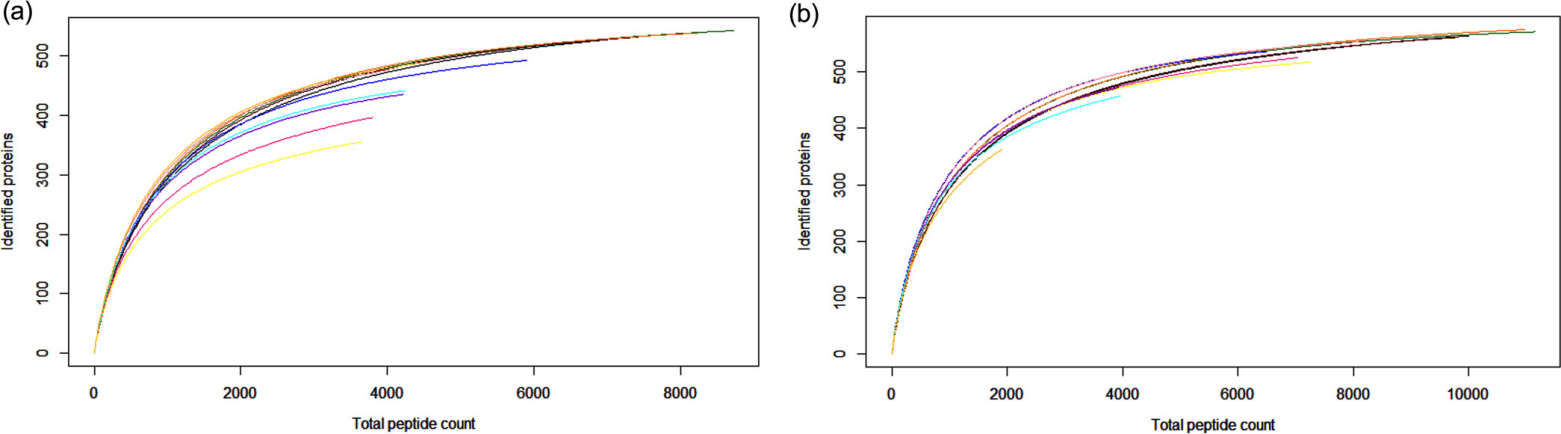
Rarefaction curves for CLE proteins identified from Malpighian tubule (a) and ovaries (b) dissected from *R. sanguineus* ticks. Each curve represents the cumulative sum of identified proteins and each sample replicate is distinguished by a different line color.

The data set included three biological replicates for every organ (Mt and Ov), each having three technical replicates (overall six samples covered by 18 independent reads). Rarefaction curves of the peptide spectral coverage of each sample replicate reached saturation, confirming that LC MS-MS sampling effort was sufficient (Fig. 1). According to the annotated protein coding of CLE in Rhipicephalus sanguineus (19), the coverage of individual organ proteome was 80% (614/764) for Mt, and 75% (580/764) for Ov. The complete list of proteins identified with peptide spectrum matches and relative abundance indexes in all 18 data sets are accessible in Table S1 in the supplemental material.

**FIG 2:**
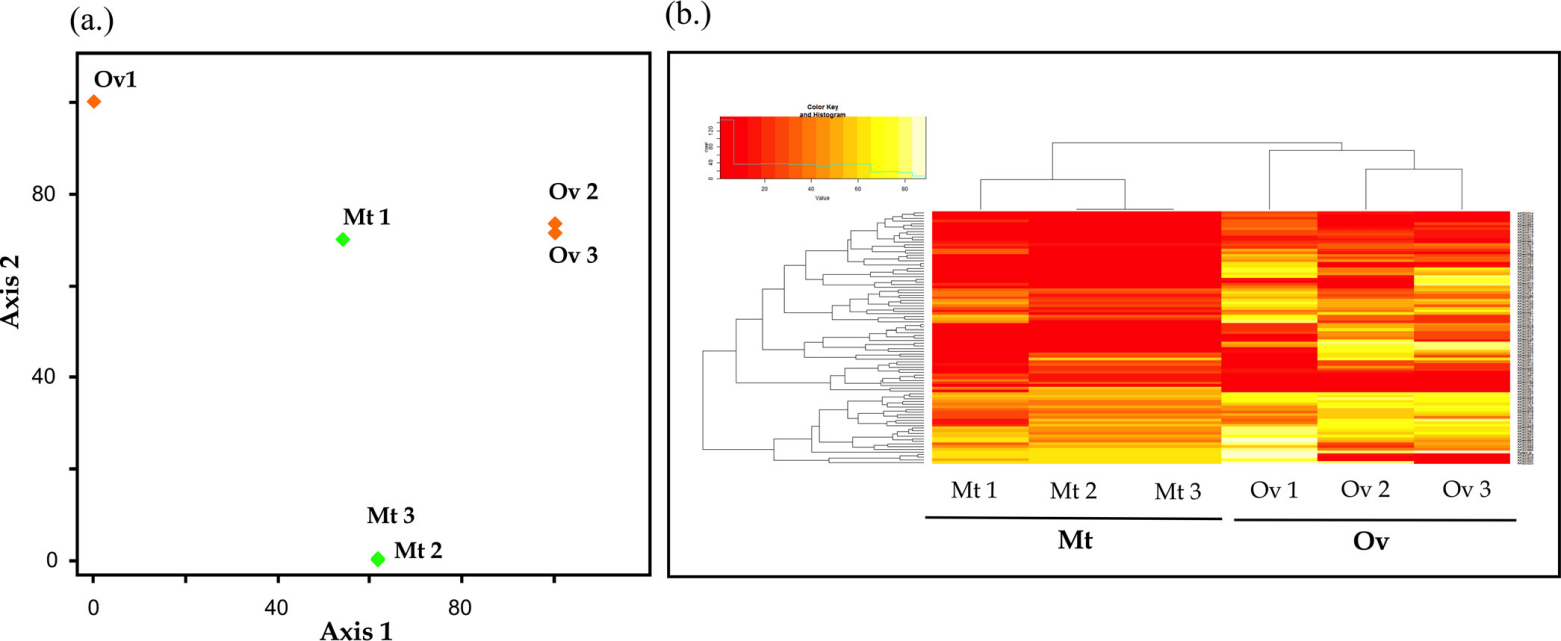
Discriminating CLE proteomics data. (a) PCA scatterplot of axis1 and 2 is based on the cumulative values of replicates of respective organs. (b) Heatmap of differential abundance expressed CLE proteins from Mt and Ov.

Principal-component analysis (PCA) clearly separated samples according to their organ source (Mt and Ov), but also pointed to variation originating from specific samples (sample 1 versus samples 2 and 3; Fig. 2a). These differences probably arise from the fact that samples 2 and 3 were collected, processed and shipped to MS analyzed simultaneously in contrast to sample 1 which may have introduced a certain degree of variability. However, the protein concentration of the three samples was similar (Table 1), implying that extraction procedure was reproducible for the three different samples. In agreement with PCA results, hierarchical clustering of protein abundance showed that Mt and Ov samples were clustered apart (Fig. 2b). Similarly, the heatmap indicated distinct protein profiles between the organs.

**TABLE 1:** Detailed information about sample IDs, protein concentration and sample volume involved in the experiment

Buffer	Protein (μg)	Sample vol (μl)	Concentration (μg/μl)	Technical replicate	Biological replicate (pooled, laboratory females)	Sample ID
0.25% Rapigest, 50 mM ammonium bicarbonate, 5 mM DTT	13.55437	45	0.301208	Mt-1a	Mt-1	Mt-1
				Mt-1b		
				Mt-1c		
	12.56557	45	0.279235	Ov-1a	Ov-1	Ov-1
				Ov-1b		
				Ov-1c		
	10.99865	4	0.24441	Mt-2a	Mt-2	Mt-2
				Mt-2b		
				Mt-2c		
	14.00785	45	0.311286	Ov-2a	Ov-2	Ov-2
				Ov-2b		
				Ov-2c		
	10.99865	45	0.244414	Mt-3a	Mt-3	Mt-3
				Mt-3b		
				Mt-3c		
	11.80652	45	0.262367	Ov-3a	Ov-3	Ov-3
				Ov-3b		
				Ov-3c		

### Differential protein abundance between organs.

**FIG 3:**
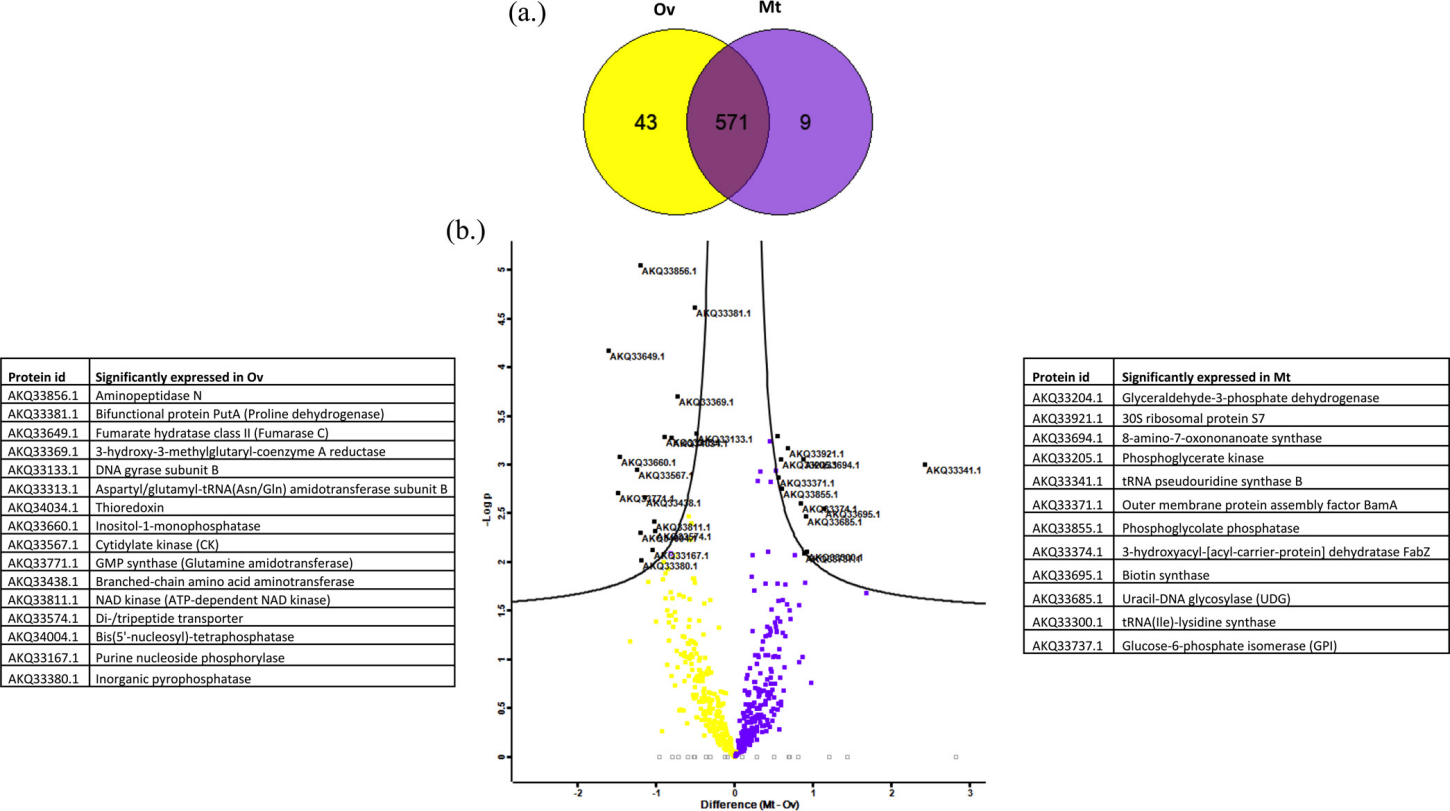
(a) Venn diagram representing the number of unique and shared proteins between Malpighian tubule (Mt) and ovary (Ov) of CLE. (b) Volcano plot of differentially abundant CLE proteins at 0.1 false-discovery rate (FDR) between Mt and Ov. The plot was generated using log_2_ fold change versus *P* values (Student's *t* test between replicate measurements). Differentially abundant proteins in violet and yellow squares represent Mt and Ov, respectively. No significant changes are shown as empty squares. The FDR cutoff is indicated by black lines and the prominent significant proteins of each organ is highlighted in black squares. Significantly, abundant proteins in each organ are listed in the adjacent tables.

Altogether, our shotgun proteomics approach identified a total of 580 and 614 CLE proteins in Mt and Ov tissues, respectively. Of these, 571 were common to both organs, and nine and 43 proteins were found only in Mt or in Ov respectively (Fig. 3a) (Table S1 in the supplemental material). Relative abundances of *Coxiella* proteins between the two organs are depicted Fig. 3b, along with the lists of 16 Ov and 12 Mt significantly differentially abundant proteins. Full information on the protein relative abundances and *P* values are provided in Table S2.

**FIG 4:**
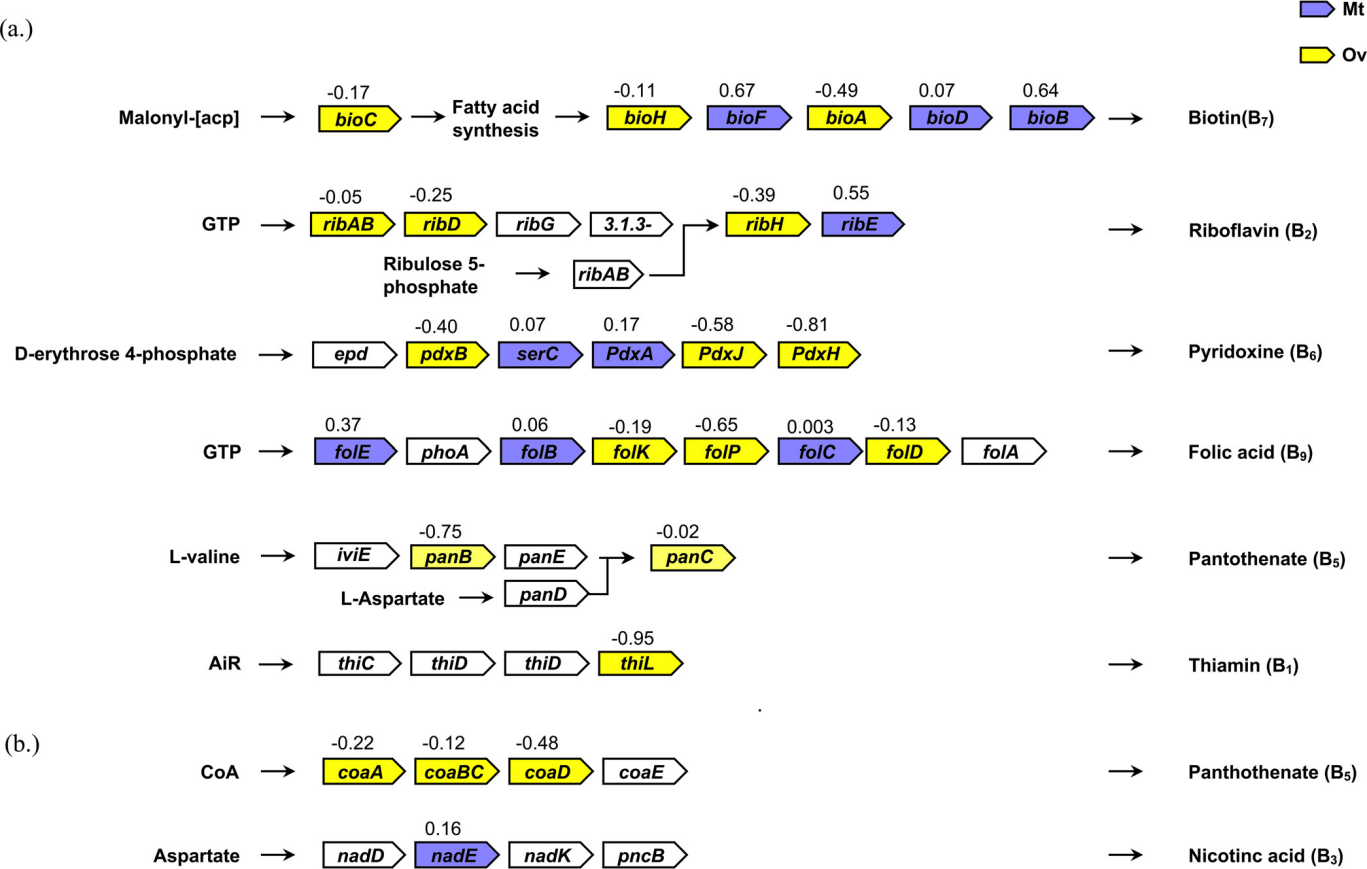
Biosynthetic metabolic pathways were highlighted based on specific abundance of CLE proteins for (a) B vitamins and (b) cofactors. Violet boxes represents Malpighian tubules (Mt), yellow indicates the ovaries (Ov) and the empty boxes are not found in both the organs. The values mentioned above the boxes indicate the log_2_ fold change differences of representative proteins.

Considering the suggested production of B vitamins and co-factors, and potentially l-proline production by CLE (19), we have manually curated proteins involved in these biosynthesis pathways and relevant differentially expressed proteins and mapped them on to their respective metabolic pathways (Fig. 4). Mt (+ values) and Ov (−values) contain one or more B vitamin proteins except for panthothenate and its co-factors, thiamine and nicotinic acid. In the Malpighian tubules, significantly expressed proteins include BioF and BioB (0.67- and 0.64-fold, respectively). The other notably expressed protein FolE (0.37-fold). Aside from BioF, BioB, and FolE, which are enzymes involved in biotin and folate synthesis respectively, other B vitamin and co-factor synthesis pathway proteins were under expressed, including BioD (biotin), RibE (riboflavin), SerC, PdxA (pyridoxine), FolB, FolC (folate), and NadE (niacin). Among the thiamine pathway, ThiL protein was expressed (–0.95-fold) only in Ov and no protein was expressed in Mt for thiamine pathway. Proteins relevant for proline metabolism were also under expressed in Mt such as ornithine cyclodeaminase (0.2-fold) and proline-tRNA ligase (0.01-fold).

In the ovaries, PpnK and PanB relevant for NADP+ and panthethonate synthesis respectively, were significantly expressed (−1.02 fold and −0.75, respectively), and notably expressed other proteins were PdxJ and PdxH (pyridoxine), FolP (folate), and CoaD (panthethonate). Other B vitamin and cofactor synthesis pathway proteins were under expressed, including BioA, BioC, BioH (biotin), RibD, RibH, RibAB (riboflavin), PdxB (pyridoxine), FolD, FolK (folate), PanC, CoaA, and CoaBC (pantothenate). Among the thiamine pathway, ThiL protein was expressed (−0.95-fold) only in Ov and no protein was expressed in Mt for thiamine pathway. However, protein related to proline metabolism, PutA was significantly over expressed (−0.5-fold).

Among transporter proteins, we detected low-level abundance of riboflavin transporter protein in Mt and significant abundance of di/tripeptide transporter protein in the Ov. Apart from transporter proteins, the relative abundances of chaperone proteins DnaK (HSP70), HtpG and small heat shock proteins were observed at 0.18-, 0.08-, and 0.07-fold, respectively. However, the Ov heat shock proteins, GroS and GroES, were present in high abundances (-0.5 fold).

### Gene ontology analysis.

**FIG 5:**
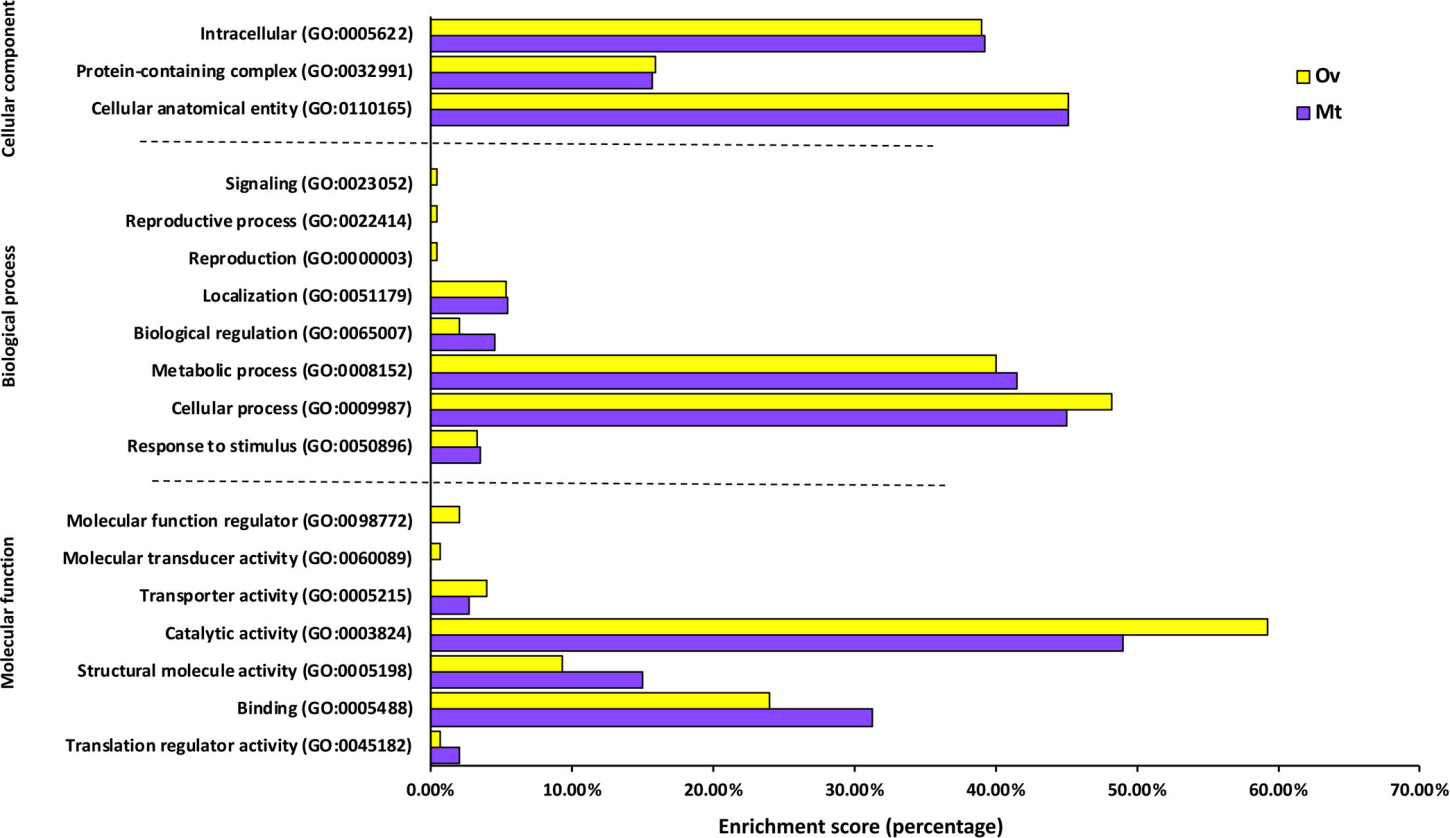
The functional classification of the differentially abundant proteins by Gene ontology analysis based on the molecular function, biological process and cellular component categories. Categorization of the ordination is based on number of genes in each category.

GO analysis of the differentially abundant proteins found significantly enriched functional groups in all major categories (Fig. 5). In the biological process group (BP) reproductive processes (GO:0022414; GO:0000003), were found exclusively in Ov-enriched proteins. Specific CLE functions in Ov were also found only in the molecular function group (MF) including molecular function regulator (GO:0098772) and molecular transducer activity (GO:0060089). These functional associations support our hypothesis that CLE act in ovaries to promote reproduction and developmental processes. In contrast, overall, functional categories in the Mt display non-exclusive but measurable increases in proteins involved in localization (GO:0051179), biological regulation (GO:0065007), metabolic process (GO:008152), binding (GO:0005488), structural molecular activity (GO:0005198), translation regulator activity (GO:0045182). This suggests that CLEs evolved specialized functions specific to the cellular environment found in Mt cells, for example, the high osmotic pressure in this organ due to its secretory activity (20).

### Metabolic mapping.

To gain a deeper understanding of the GO categorical differences described above, we mapped KEGG ortholog identities of the differentially abundant proteins onto their respective metabolic pathways onto, using iPath 3.0 web-based tool (see Fig. S1 in the supplemental material). Overall, KEGG orthologs mapped to pathways such as metabolism of carbohydrates, cofactors and vitamins, energy, nucleotides, lipids, and amino acids. Clear differences in the coverage of specific pathways between the two tissues were observed and in general, CLE proteins in the ovary displayed more complete coverage within specific pathways including lipid and glyoxylate metabolism. The main differences between the metabolic maps of the two symbiotic organs were the pathways of vitamins, cofactors and fatty acid metabolism. The proteins of CLE in Ov mapped to the metabolism of vitamins and cofactors pathways, while the Mt metabolic map showed incomplete pathways. Besides, vitamins, enzymes in the fatty acid and glyoxylate metabolism pathways were found to be abundant in Ov compared with Mt (FabD, FabV, FabF and FabH versus FabG and FabZ; Mdh, AldA, Gph, and AcnA, versus GltA, respectively). This suggests that CLEs may either utilize this pathway in the ovaries as energy source for its own proliferation, or to support the developing oocytes of the host.

## DISCUSSION

In the present study, we used comparative proteomics to reveal the metabolic potential of CLE in the major symbiotic organs, the Malpighian tubules and the ovaries, in Rhipicephalus sanguineus unfed female ticks. Our data are of high quality and provide substantial coverage of the entire CLE proteome, with 80% in Ov and 75% in Mt. Most CLE proteins were shared across both organs including housekeeping proteins for homeostasis and viability, including many of the B vitamin synthesis enzymes thought essential for host fitness (17). However, differential protein abundance measurements in each tissue suggest CLE-specific functions in each organ. In particular, transporter and heat shock proteins were found to have differential amounts in each organ.

The Malpighian tubules function in excretion of nitrogenous products and osmoregulation with surrounding hemolymph (9). A likely hypothesis is that CLE recycle hemolymph metabolites to synthesize novel compounds such as B vitamins. Although we detected vitamin B synthetic pathway enzymes in Mt, they are significantly less abundant when compared to ovaries. Nevertheless, of all the relevant enzymes in the biotin synthesis pathway, BioF and BioB were significantly more abundant in Mt. The genomic evidence suggest that in *R. turanicus*, CLE participate by providing biotin (B7) usually not obtainable from the tick diet (17). Symbionts of blood-feeding arthropods produce a core of B vitamin supplement for their hosts, which include biotin (reviewed in Ref. 2). Thus, although most B vitamin synthesis pathways are reduced in Mt, the core B vitamin, biotin, is elevated and perhaps also transported to the host.

Other than B vitamin synthesis proteins, we also detected differential abundance of transporter and stress related heat shock proteins in the Mt. The gene ontology analysis showed differential detection of proteins in the stimulus response group. These protein classes are related to DNA repair mechanism. Of interest are Ung, uracil-DNA glycosylase, and SpoT GTP, pyrophosphokinase. Ung excises DNA uracil residues that arise either by misincorporation of dUTP or by spontaneous deamination of cytosine residues in order to prevent mutagenesis (21). Considering the evolutionary path of obligatory endosymbiont genomes toward pseudogenization and length reduction (22), CLE may synthesize Ung for maintaining a functional genome. SpoT is a bifunctional enzyme that has both (p)ppGpp synthetic and hydrolytic activities that integrate stress signals especially during iron limitation (23). The increased levels of (p)ppGpp leads to impaired transcription and translation (24). Thus, SpoT may be relevant when the tick host is unfed and is under iron limitation and further cellular stress which impose DNA repair gene expression.

Besides the presence of aforementioned essential proteins, DnaK (Hsp 70) and small heat shock proteins were observed in Mt. In general, heat shock proteins (Hsp) are expressed specifically in bacteria and in the organelles of eukaryotic cells (25). When ticks are off host, they can undergo extended starvation and are exposed to various environmental stressors (26, 27). These may also affect CLE, and Hsp expression can play a significant role in stabilizing its metabolic activity. Thus, the stress related proteins expressed in the Mt, will maintain homeostasis under cellular stress factors including nutrient deprivation and osmotic pressure. We also detected the enzyme ornithine cyclodeaminase, which converts l-ornithine to l-proline. In general, bacterial species can synthesize l-proline in response to osmolarity changes (28), and perhaps a similar mechanism is adapted by CLEs to cope with the osmotic stress in the Mt cells. Further support to the osmo-protective function of CLE in Mt is the detection of proton membrane transport proteins. Detected only in the Mt, SspA, Stringent starvation protein may enable CLEs to regulate osmotic pressures and maintain balanced pH via inward transportation of hydrogen ion. Detection of transporter proteins may also support a metabolic collaboration with the host by transporting ions and essential amino acids. For example, the riboflavin transporter protein may use ATP-derived energy to export riboflavin against the concentration gradient (29), presumably from CLE intracellular environment to the host. Overall, the CLEs in Mt tends to produce the essential core B vitamins and heat shock proteins for its self-stabilization, or it may support the tick host for its survival during nutrient deprived condition.

In the ovaries a greater number of CLE proteins were observed compared to Mt (614 versus 580). This increase may be related to a requirement imposed by the known transovarial transmission of the endosymbiont via the egg (4, 5, 8, 30, 31). It is unclear if CLEs in Ov provide B vitamins to the tick host and additional study is needed in this area. However, consistent with transovarial transmission and the expected need for proliferation and growth during oogenesis, proteins involved in development and reproduction were detected in Ov. In particular, enrichment of ZapD, DedD and YgbF proteins, are known components of cell division processes in E. coli (32, 33). Apart from B vitamins and proliferation related proteins, PutA, proline dehydrogenase, which is responsible for l-proline catabolism, especially the conversion of proline to glutamate, is highly abundant in the ovaries. PutA is ubiquitous in bacteriomes (34, 35) and in insect hemolymph (36). Its expression is involved in energy metabolism of *Wigglesworthia* in tsetse flies (37) and synthesis of peptidoglycan (38). PutA enables bacteria to utilize proline as a source of carbon and nitrogen when grown under poor nutrient conditions and for initiating bacterial proliferation (39, 40). Similarly, CLEs may utilize proline by PutA during starvation conditions to proliferate in Ov.

Apart from the above proteins, CLEs also exhibit enrichment of the stress response chaperone proteins, GroS GroES, which is a co-factor for GroEL. During the downstream analysis of proteomics results, we deliberately eliminated GroEL due to its extreme overexpression, as we mentioned in the materials and methods. This can be correlated with CLEs production of GroES and GroS in higher concentrations to assist in the folding of conformationally damaged proteins and thus mitigates the negative effects of deleterious mutations occurring due to genome erosion (41).

Most of the transporter proteins detected in the Ov belong to ABC (ATP-binding-cassette) transporter. A previous genomic study of B. aphidicola (42, 43) demonstrated export of ATP from endosymbionts to host cells. Thus, the data suggests a possible linkage between energy production in Ov by CLEs and its exporting to host.

CLEs is omnipresent in both the organs, the expression of their proteins may depend on the requirement for their own survival (such as adapting to stressful cellular environment and proliferation) or to their host tick (production of core B vitamins). In agreement with the genomic evidence of CLEs (17–19) we have not observed complete synthesis pathways for B vitamins in both the organs, and we could detect l-proline relevant enzymes which seem to be essential both for CLE maintenance and also for the tick. To the best of our knowledge, there are no other predominant bacteria in Mt and Ov of *R. sanguineus* ticks (5, 12), although maternal transmission of *Rickettsia* via the tick ovaries is possible (44). The ticks used in our study were from the same colony of ticks used in our previous study (12), confirming that they are free of *Rickettsia*. Moreover, routine testing of *R. sanguineus* ticks in the lab via various methods (5, 12, 44), established the dominancy of CLEs.

## CONCLUSION

Organ-specific comparative proteomics analysis may reveal the metabolic functions involved in maintaining symbiosis and homeostasis under stressful conditions. CLEs response to nutritional starvation of *R. sanguineus* ticks by modifying protein expression in Mt and Ov. In each organ, CLE has its unique proteins abundance which may depend on its own and/or the host requirements. CLE proteins expressed in Mt may be related to self-metabolic functions and to stress response, while CLE proteins expressed in the ovaries may have function related to self-proliferation to be vertically transmitted by the host. We have used hosts under starvation; thus our results are limited to this feeding state. Additional limitation to our study is the low quantity of bacterial protein that can be retrieved from mixed symbiont-host tissues dominated by host proteins, as well as limited number of membrane-imbedded proteins. To further understand the function of CLE in symbiotic organs of ticks, different feeding and developmental states need to be studied, and better protocol for the enrichment of symbionts proteins should be developed.

## MATERIALS AND METHODS

### Separation and collection of tick organs.

Unfed females of Rhipicephalus sanguineus were obtained from a tick colony as previously described (12). The ticks were externally rinsed in a mild detergent solution (1% Alconox, USA) for 1 min, surface sterilized in 1% sodium hypochlorite for 1 min, and finally washed three times in sterile saline. Dissections were performed on ice under a stereomicroscope using sterile forceps and microdissection scissors as previously described (5). Briefly, live ticks were anesthetized by chilling on ice, affixed on wax and covered with 50–150 μl of ice-cold phosphate-buffered saline (PBS, Biological Industries, Israel) containing protease inhibitors (MSsafe, Sigma, MO, USA). Organs were removed and washed in 100 μl of ice-cold PBS and immediately frozen (−80°C) in 25 μl of PBS. Care was taken to remove only the distal part of each Malpighian tubule containing CLEs (5). Ovaries were removed intact. Protein was extracted from 20 individual samples of Mt and Ov per sample as described next, in three biological replicates (Table 1).

### Protein extraction.

Frozen samples were thawed on ice and centrifuged at 20,000 g for 15 min at 4°C to pellet organs and any suspended bacteria. The supernatant was decanted and 50–100 μl of 0.25% Rapigest surfactant (Waters, Milford, MA, USA) in 50 mM ammonium bicarbonate buffer was added. Organs were subsequently disrupted by sonication on a Vibra-Cell VCX 750 machine (Sonics & Materials Inc., CT, USA) equipped with a cup-horn probe: 3 bursts at full power for 30 s each and incubated at 60°C for 30 min to complete tissue lysis.

Total protein content of each sample was determined by the Pierce Bicinchoninic Acid protein assay kit (Product #23227, Thermo Scientific, IL, USA) on a NanoDrop 2000 spectrophotometer (Thermo, MA, USA) according to the manufacturer’s instructions. All samples were measured in triplicate. Calibration curves were generated with Bovine Serum Albumin (BSA, Sigma, MO, USA).

### Proteome analysis by mass spectrometry (MS/MS).

All LC-MS/MS (liquid chromatography with tandem mass spectrometry) analyses were performed at the University of California San Francisco Biomedical Mass Spectrometry and Proteomics Resource Center (http://msf.ucsf.edu).

Dithiothreitol (DTT; Sigma, MO, USA) was added to the samples to a final concentration of 5 mM, and the samples were incubated at 60°C for 10 min, then alkylated with 20 mM iodoacetamide (1 h at room temperature), adjusted to pH 8 with 100 mM ammonium bicarbonate, and digested with trypsin (Promega, Fitchburg, WI, United States) added at a 1:20 (w:w) ratio (overnight at 37°C). The digests were acidified to 10% formic acid, incubated 30 min at room temperature and cleared by centrifugation at 10,000 g for 5 min. Peptides in the supernatant were extracted using a 100 μl C18 OMIX tip (Agilent, Santa Clara, CA, United States), according to the manufacturer’s instructions. 5 μg aliquots of the tryptic digest of each sample were injected in 50 cm EasySpray columns (Thermo Scientific) and analyzed in triplicate (technical replicates of the samples referenced in the results) using 310 min reversed-phase (RP) gradients in a nanoACQUITY UPLC (Ultra Performance Liquid Chromatography) system (Waters, Milford, MA, United States) coupled to a QExactive mass spectrometer (Thermo Fisher Scientific).

Peak lists were generated using PAVA in-house software (45). All generated peak lists were searched against the *Ixodes* subset of the UniProt database (UniprotKB 2017.11.01) plus the published genome of *Coxiella* from *Rhipicephalus turanicus* (CRt; CP011126), using Protein Prospector (46), with the following parameters: Enzyme specificity was set as Trypsin, and up to 2 missed cleavages per peptide were allowed. Carbamidomethylation of cysteine residues was allowed as fixed modification. N-acetylation of the N-terminus of the protein, loss of protein N-terminal methionine, pyroglutamate formation from peptide N-terminal glutamines, oxidation of methionines were allowed as variable modifications. Mass tolerance was 10 ppm in MS and 30 ppm in MS/MS. The false positive rate was estimated by searching the data using a concatenated database, which contains the original database, as well as a version of each original entry where the sequence has been randomized. A 1% FDR was permitted at the protein and peptide level.

The peptide spectrum matches (PSMs) for *Coxiella* proteins were counted and exported to calculate normalized abundance indexes by normalizing these values by molecular weight and total *Coxiella* PSMs in that particular MS run. Normalized abundance indexes were then used to compare protein levels between tissues as described next.

### Data analyses and visualization.

The peptides that were associated with the tick host were removed from further analyses that focused on the CLE proteome solely. To assess the differences and similarities between Mt and Ov and to understand the reproducibility of the three biological samples, normalized protein data (relative abundance indexes) of each biological repeat were first analyzed using Perseus v.1.6.0.7. The data were annotated and log_2_ transformed (47) to determine which proteins were significantly enriched in any experimental group. Thereafter, for sample comparison, we used PCA (PCORD v.6), and a heatmap analysis (R package ggplot2 v.3.5.3). In the data set, protein GroEL (AKQ33233) was removed from further analysis due to extremely high spectral coverage ca. 5-10 fold, which mask over the rest of the identified protein in our sample set. GroEL is a known predominant chaperonin found in many endosymbionts (48).

Using all the three biological repeats, unique and shared proteins between the two organs (Mt and Ov) were visualized in a Venn diagram (GeneVenn; http://genevenn.sourceforge.net/). Organs were compared using t-tests and volcano plots with the customized settings of FDR cut-off at 0.1 and S_0_ = 0.1. The statistically significant differentially abundant proteins obtained from these t-tests were used to generate volcano plots. The shape of the cut-off curve of the volcano plot was calculated by Perseus (49). The list of all proteins was then submitted to a gene ontology (GO) analysis using PANTHER classification system (http://www.pantherdb.org/) v.15.0 (50). Through this, the proteins were categorized in the main three GO domains: biological processes, molecular functions, and cellular components. To further assess the complementarity system of CLE, the reactions encoded in the different physiological states, protein encoding genes were imported into the interactive metabolic pathway explorer iPath3.0 (https://pathways.embl.de/ipath3.cgi) for visualization of metabolic and cellular pathways (51).

### Data availability.

The mass spectrometry proteomics data have been deposited to the ProteomeXchange consortium (52) via the PRIDE partner repository with the data set identifier PXD026817. We have the data set identifier as stated.
