# Supplementary material for: Comparative Proteomics of Coxiella like Endosymbionts (CLEs) in the Symbiotic Organs of Rhipicephalus sanguineus Ticks
Source: Microbiol Spectr. 2022 Jan 12;10(1):e01673-21. doi: 10.1128/spectrum.01673-21 (PMC8754119; doi:10.1128/spectrum.01673-21)
Supplement: SUPPLEMENTAL FILE 3 — Supplemental material. Download SPECTRUM01673-21_Supp_1_seq7.pdf, PDF file, 5.5 MB [file spectrum01673-21_supp_1_seq7.pdf]

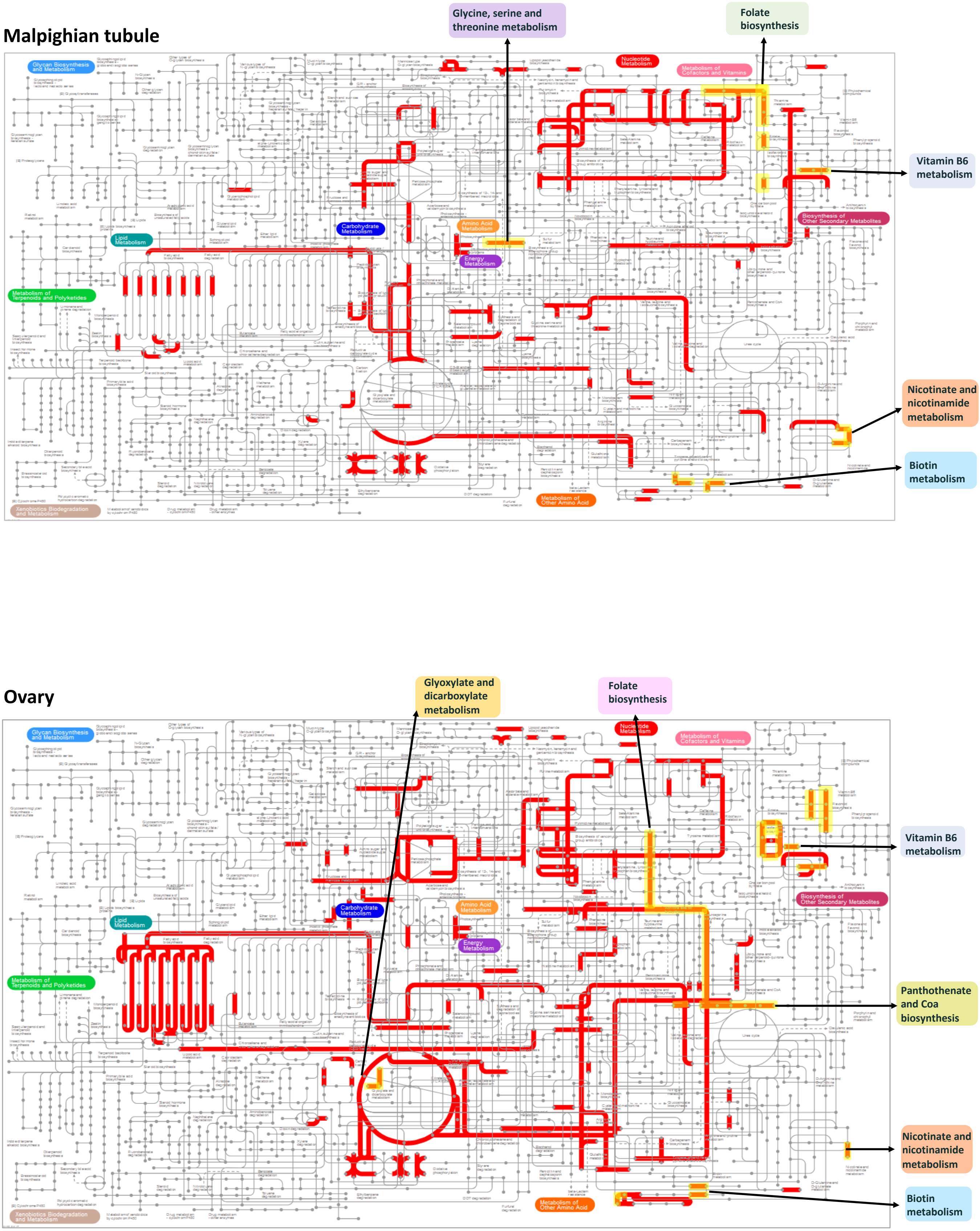

Figure S1: Overview of interactive metabolic pathways based on CLE proteins presence in Mt (top) and Ov (bottom). The nodes colored ni red represent pathways of all enzymes or proteins annotated from genomic data and the B-vitamin pathways in Mt and Ov are highlighted in yellow. The metabolic pathways were mapped using ipath3.0.
